# The potential of SiK® fertilization in the resilience of chestnut plants to drought - a biochemical study

**DOI:** 10.3389/fpls.2023.1120226

**Published:** 2023-06-26

**Authors:** Andreia Carneiro-Carvalho, Teresa Pinto, José Gomes-Laranjo, Rosário Anjos

**Affiliations:** CITAB-Centre for the Research and Technology of Agro-Environmental and Biological Sciences, and Inov4Agro – Institute for Innovation, Capacity Building and Sustainability of Agri-food Production University of Tras-os-Montes and Alto Douro (UTAD), Vila Real, Portugal

**Keywords:** *Castanea sativa*, drought, oxidative damage, silicon, tolerance, antioxidant activity, lipid peroxidation, water stress

## Abstract

Silicon is an essential mineral nutrient, that plays a crucial role in the metabolic, biochemical, and functional mechanisms of many crops under environmental stress. In the current study, we evaluated the effect of SiK^®^ fertilization on the biochemical defense response in plants exposed to water stress. *Castanea sativa* plants were fertilized with different concentrations of potassium silicate (0, 5, 7.5, and 10 mM of SiK^®^) and exposed to a non-irrigation phase and an irrigation phase. The results indicate that silicon promoted the synthesis of soluble proteins and decreased the proline content and the oxidative stress (reduced electrolyte leakage, lipid peroxidation, and hydrogen peroxide accumulation) in tissues, due to an increase in ascorbate peroxidase, catalase, and peroxidase activity, which was accompanied by the rise in total phenol compounds and the number of thiols under drought conditions. This study suggests that exogenous Si applications have a protective role in chestnut plants under water deficit by increasing their resilience to this abiotic stress

## 1 Introduction

Climate change has led to greater drought, which causes problems in chestnut trees affecting growth and fruit production, because of low water availability in the soil during this period. The use of silicon in agriculture has proven to be very useful in several cultures; it is a mineral that positively affects the development of plants and acts in the deleterious effects of abiotic stresses (Rea et al., 2022). Silicon appears as a mitigating strategy that modulates plant metabolism and interferes with physical and biochemical mechanisms, promoting resistance to the devastating effects promoted by hydric stress (Mello et al., 2022). The role of Si in plant development is associated with the absorption of monosilicic acid (H_4_SiO_4_) through the root, by a passive process regulated by transpiration through the xylem or by an active process, through specific transporters located in the plasmatic membrane of root cells. Many authors explained that Si is accumulated inside the plant tissues, as amorphous silica (SiO_2_·*n*H_2_O), increasing polymerization in the intercellular system and below the cuticles, creating a barrier, that contributes substantially to strengthening the plant structure and increasing their resistance mechanics. Recent studies showed that plants develop a well-optimized Si transport system including several transporter proteins, such as low silicon1 (Lsi1), low silicon2 (Lsi2), low silicon3 (Lsi3), and low silicon6 (Lsi6), in specific subcellular sites, along with the expression profile that creates a precisely coordinated network between these transporters, which facilitates the Si absorption and accumulation (Gaur et al., 2020; Prado, 2020; Mir et al., 2022).

Silicon is a mitigation agent for several plant species, and the benefits of abiotic stress are divided into two groups: 1) physical and physiological mechanisms and 2) biochemical and enzymatic mechanisms. Physical and physiological mechanisms are related to the accumulation of Si in the cell walls, improving the architecture of the plants and reducing water loss by the leaves. In addition, Si keeps the physiological systems and photosynthetic processes under water deficit, reducing transpiration, improving osmotic adjustment, reducing water demand by plants, decreasing stomatal opening, and improving productivity (Souri et al., 2021; Rea et al., 2022; Naaz et al., 2023). On the other hand, more recent studies reinforcing these mechanisms explained that Si stimulates an increase in the leaf water status of crops, by enhancing the osmotic driving force; promotes the activity of aquaporins, modifying root growth; and increases the root/shoot ratio, regulating evapotranspiration and improving the root hydraulic physiochemistry, contributing to increasing the plant productivity (Chen et al., 2018; Cassel et al., 2021; Rossetti, 2022). Biochemically, Si induces the plant’s own defense mechanisms by activating various defense strategies, including the synthesis of phenolic compounds and the production of lignin, suberin, and callose in the cell wall, reducing the lipid peroxidation and promoting the accumulation of hydrogen peroxide (H_2_O_2_) (Kim et al., 2017; Hasanuzzaman et al., 2018; Navarro, 2020). Furthermore, Si acts in enzymatic mechanisms activating the enzymatic complexes, relieving oxidative stress on functional molecules and membranes, and increasing important antioxidant enzymes (Kim et al., 2017). According to many authors, the essential role of Si against water deficit is also related to the activity of non-enzymatic antioxidants, such as reduced glutathione (GSH) and ascorbate (AsA), which in turn decrease the level of lipid peroxidation and lipoxygenase (LOX) activity, relieving oxidative stress caused by ROS in plants under water stress (Kim et al., 2017; Hasanuzzaman et al., 2018). The combination of physical and biochemical defenses by silicon fertilization in plants promotes significant resilience against drought.

Previous studies carried out about the application of Si in chestnut trees demonstrated that Si-treated plants at concentrations of 7.5 and 10 mM of SiK promote higher resilience in adverse conditions (Navarro, 2020). Taking into account the existence of few studies about the role of Si in the resistance of the chestnut tree to drought, it was decided to carry out the present study to evaluate the potential of Si fertilization in promoting the resistance of the chestnut tree to water stress, namely, in the biochemical and enzymatic mechanisms, in order to help chestnut producers during the increasingly frequent drought periods (Carneiro-Carvalho et al., 2020; Carneiro-Carvalho et al., 2021).

## 2 Material and methods

### 2.1 Experimental layout and treatments

**Figure 1:**
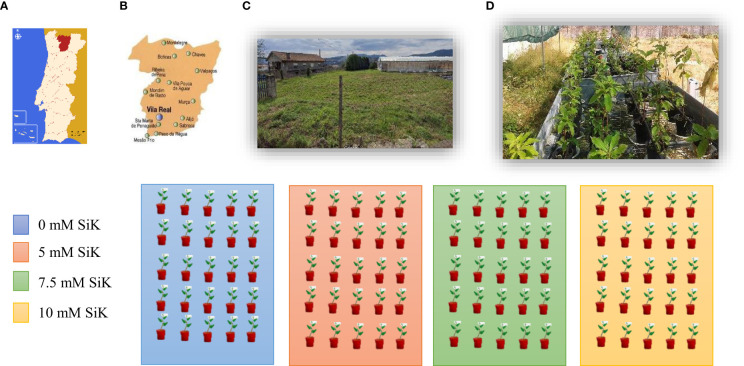
Experimental design. **(A)** Schematic map of the Portugal-Vila Real indicating the locality where the study was developed. **(B)** Map of Vila Real city. **(C)** Satellite image showing the arrangement of the study experimental in Folhadelha inside Quinta dos Prados - University of Tras os Montes and Alto Douro, where the assay took place. **(C)** Picture showing the experimental plan development over two years. **(D)** Schematic representation of the experimental plan: four benches, one per treatment (0, 5, 7.5, and 10 mM SiK), thirty plants per box, and one cultivar (*Castanea sativa* cultivar Sousã). All plants were grafted onto the same rootstock [70% turf and 30% perlite at a ratio of 3:1 (v/v)].

The experiment (**Figure 1**) was conducted in 2015 and 2016 in a field in Folhadela belonging to the University of Trás-os-Montes and Alto Douro, Vila Real (41°17′20″N, 7°44′0″W) in the north of Portugal. The area is characterized by a humid subtropical climate (www.it.climatedata.org) with hot and dry summers. In both assays, plants belonging to the specie *Castanea sativa*, 4 years old, having been grafted at 2 years old with a Portuguese cultivar called Sousã, were used. This is a cultivar that presents good compatibility with the *C. sativa* species, in addition to being well adapted to the climatic conditions of the region of Trás-os-Montes, Portugal. The chestnut plants were planted in 6-L plastic pots filled with 70% of turf and 30% of perlite at a ratio of 3:1 (v/v). Chestnut plants were fertilized with increasing concentrations of potassium silicate (SiK^®^) (0, 5, 7.5, and 10 mM of SiK^®^) in two ways, directly in the soil (50 ml) and on the leaves (30 ml) with foliar application, to reinforce the accumulation of silicon in tissues and increase the resilience of plants. In the present study, the plants were submitted to annual SiK^®^ applications between 2014 and 2016. The concentrations of SiK^®^ were selected from the results obtained in previous studies of chestnut plants (Carneiro-Carvalho et al., 2020; Carneiro-Carvalho et al., 2021). Each treatment containing 30 plants and was set 12 replicates per treatment; in the study, a total of 120 plants were used. Water stress has been induced in the study by turning off the automatic irrigation system. All plants were submitted to a first phase without irrigation by turning off the automatic irrigation system (non-irrigation phase, NIP) to induce water stress. When the first plants reached their threshold of water potential, −2.0 MPa, having verified the death of several plants for this period with no water supply being provided, the irrigation was opened (second phase—irrigation phase, IP), to evaluate the recovery capacity of the plants after the water stress period. A drip system (4 L/drip/plant) was installed to ensure irrigation. During the irrigation phase, water was applied daily for 15 min.

The samples used for the determination of the different parameters were the leaves, which were collected after their exposure to water stress, during both the NIP and after the IP, when plants reach the higher water potential.

### 2.2 Determination of the content of total phenol compounds

Four discs (8 mm diameter) from each leaf were used, and the extraction was done using 10 ml of 80% acetone (v/v). The total phenol compounds (TP) were quantified using 5 ml of the acetonic extract according to the Folin–Ciocalteu procedure (Singleton and Rossi, 1965). The absorbance was measured in the spectrophotometer at 795 nm, with six replications per treatment (*n* = 6).

### 2.3 Soluble sugars and starch quantification

The extraction of total soluble sugars (SS) was done in 80% ethanol (v/v), through the colorimetric method (Irigoyen et al., 1992). The absorbance of the samples was determined at 625 nm. Starch (ST) was determined from a pellet of SS, with the extraction being carried out in 30% perchloric acid (v/v) and quantified at 625 nm (Rose et al., 1991). Glucose was used as a standard for the SS and ST determinations, with six replications per treatment (*n* = 6).

### 2.4 Quantification of the electrolyte leakage percentage, lipid peroxidation, and hydrogen peroxide amount

Electrolyte leakage is a method to evaluate the stability of the cell membrane to stress, which is an indicator of plant tolerance (Bajji et al., 2002). The determination of electrolyte leakage percentage (ELP) was measured using leaf discs with 8 mm of diameter (Abu-Muriefah, 2015). Leaf discs were placed in 10 ml of double-distilled water at 25°C in a shaker (100 rpm) for 24 h. Their conductivity was recorded (C1) using an electrical conductivity meter (Jenway 470 portable conductivity meter). Then, the samples were autoclaved at 120°C for 20 min and their conductivity was also recorded (C2). The percentage of electrolyte leakage was calculated according to the following formula:


ELP (%)=C1/C2

The determination of electrolyte leakage was measured in NIP and again in IP, with six replications per treatment (*n* = 6).

The lipid peroxidation (LPO) content was measured using the absorbance of the supernatants and was determined at 532 and 600 nm. The LPO was expressed as μM g^−1^ FM, by using an extinction coefficient of 155 mM^−1^ cm^−1^ (Ali et al., 2018). The quantification of LPO was measured in NIP and IP, with six replications per treatment (*n* = 6).

The H_2_O_2_ contents in the chestnut leaves were measured by the absorbance of the samples at 390 nm, and the H_2_O_2_ content was expressed as μM g^−1^ FM (Mostofa et al., 2021). The present results correspond to the leaves quantified in NIP and IP, with six replications per treatment (*n* = 6).

### 2.5 Proline content assay

The proline determination was determined through five discs (8 mm) homogenized in 3% sulfosalicylic acid, followed by centrifugation at 5,000×*g* for 20 min. The supernatant was treated with acid ninhydrin (C_9_H_6_O_4_) and 2 ml of glacial acetic acid (C_2_H_4_O_2_) and boiled for 1 h, and after cooling, 2 ml of toluene was added to each sample (Bates et al., 1973). The absorbance was determined spectrophotometrically at 520 nm, and the measurement was replicated six times per treatment (*n* = 6).

### 2.6 Thiol content assay

The determination of the thiol content was made by homogenizing a disc (8 mm) in 50 mM Tris–HCl with 20 mM of ethylenediaminetetraacetic acid (EDTA), with a pH of 8.0, and the samples were then centrifuged for 20 min at 15,000×*g*. For thiol determination, 0.25 ml of the homogenates were mixed with 0.75 ml of 0.2 M Tris–HCl, pH 8.2, and 0.05 ml of 0.01 M of 5,5′-dithiobis(2-nitrobenzoic acid) (DTNB). The absorbance of the supernatant was read at 412 nm, and the amount of thiol was calculated by using the extinction coefficient of 13.1 mM^−1^ cm^−1^ (Mostofa et al., 2021). The measurement was replicated six times per treatment (*n* = 6).

### 2.7 Quantification of the soluble protein content

Determination of the total soluble protein (SP) content was carried out using bovine serum albumin (BSA) as a standard (Bradford, 1976). The measurement was replicated six times per treatment (*n* = 6).

### 2.8 Antioxidant activity determination

Leaves were homogenized in ice-cold 0.1 M of sodium phosphate buffer (pH 6.8). The homogenate was centrifuged at 12,000×*g* for 20 min, and the supernatants were used for enzyme activity assays. The catalase (CAT) activity was measured using the Clark-type O_2_ electrode connected to an appropriate register (Wu et al., 2014). The apparatus used for measuring the release of oxygen was Oxylab Hansatech, and the activity was expressed as nmol mg^−1^ protein min^−1^. The peroxidase (POD) activity was determined with the increase in absorbance due to the oxidation of guaiacol being measured at 470 nm (Farooq et al., 2013). The POD activity was expressed as nmol mg^−1^ protein min^−1^ using the appropriate extinction coefficient (*ϵ* = 26.6 mM^−1^ cm^−1^). Ascorbate peroxidase (APX) activity was assayed through the oxidation of ascorbate and was observed by the change in absorbency at 290 nm, while the activity of APX was expressed as nmol mg^−1^ protein min^−1^ using the appropriate extinction coefficient (*ϵ* = 2.8 mM^−1^ cm^−1^) (Wu et al., 2014). The measurement was replicated six times per treatment (*n* = 6).

### 2.9 Statistical analysis

The data were subjected to an analysis of variance using StatView 5.0 (SAS Institute Inc. 1992-1998) and Statistica 8.0 (StatSoft Inc. 2007) and statistical analysis is one-way. Statistical differences among treatments were determined by the Tukey test (*P* ≤ 0.05).

## 3 Results

### 3.1 Total phenol compounds

**Figure 2:**
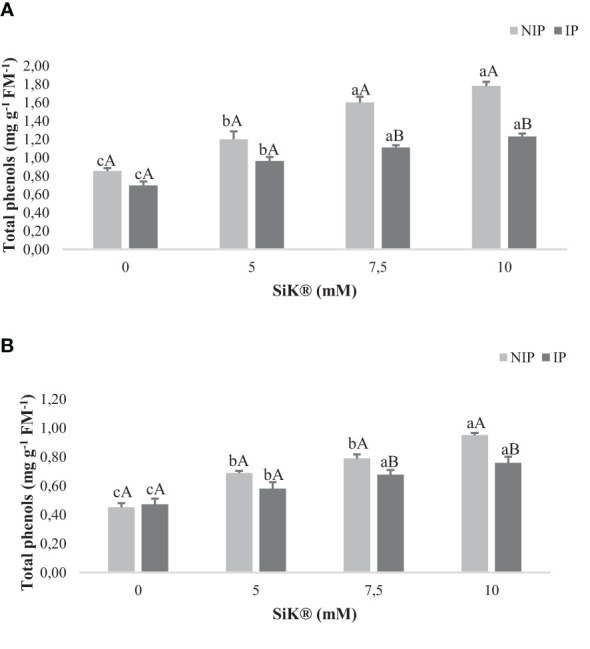
Effect of Si application (0, 5, 7.5, and 10 mM of SiK^®^) on the total content of phenol compounds (mg g^−1^ FM^−1^) of chestnut plants exposed to the non-irrigation phase (NIP) and the irrigation phase (IP) in 2015 **(A)** and 2016 **(B)**. Different uppercase letters indicate significant differences between treatments, and lowercase letters indicate significant differences between the same treatment at different water availability (NIP/IP), according to the Tukey test (*P* ≤ 0.05). The columns described correspond to the means from six repetitions and the standard error (SE).

The production of phenolic compounds is one of the strategies used by plants under environmental stress to avoid the oxidative damage caused by drought (Varela et al., 2016). As shown in **Figure 2**, the exposure to NIP caused a significant increase in TP in Si-treated plants when compared with untreated plants, approximately 111% from 0.855 to 1.780 mg g^−1^ FM^−1^ in 2015 (**Figure 2A**) and 108% from 0.451 to 0.951 mg g^−1^ FM^−1^ in 2016 (**Figure 2B**) between the 0- and 10-mM SiK^®^ treatments.

Concerning the recovery phase (IP, **Figures 2A, B**), an increase in TP was observed in Si-treated plants, with this increase being proportional to the SiK^®^ content applied to chestnut plants. Comparing NIP with IP, the data indicate that under water deficit the Si-treated plants promote a higher TP synthesis as a defense response. The Si-fertilized plants presented a higher accumulation of TP in leaf tissues under stress, 45% more from IP to NIP, while the control plants recorded only 23% in 2015 (**Figure 2A**). Similar results were found in 2016, with 25% more in Si-treated plants (10 mM of SiK^®^) and 5% in untreated plants (0 mM of SiK^®^) (**Figure 2B**). These results reinforce the positive effect of Si on the production of phenols in plants under stress conditions, suggesting that they have a beneficial role in the biochemical defense responses of chestnut plants, which promotes tolerance against water deficit.

### 3.2 Soluble sugars and starch

**Figure 3:**
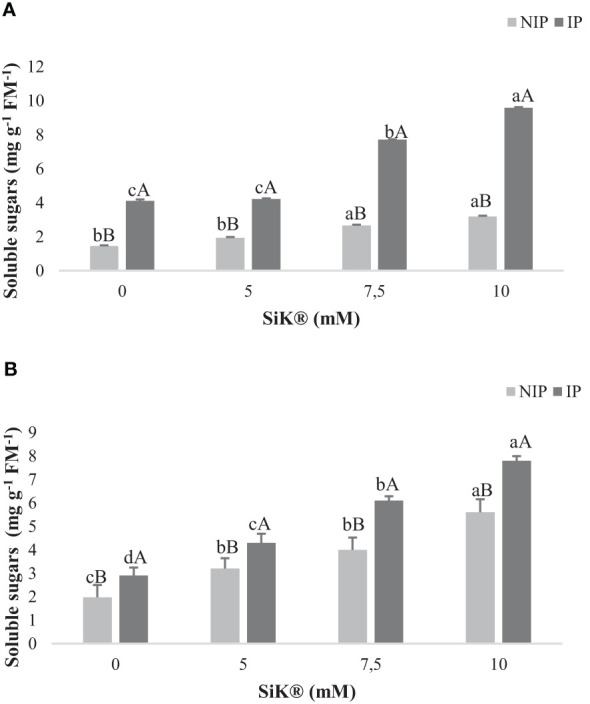
The effect of Si application (0, 5, 7.5, and 10 mM of SiK^®^) on soluble sugar content (mg g^−1^ FM^−1^) of chestnut plants exposed to the non-irrigation phase (NIP) and the irrigation phase (IP) in 2015 **(A)** and 2016 **(B)**. Different uppercase letters indicate significant differences between treatments, and lowercase letters indicate significant differences between the same treatment at different water availability (NIP/IP) according to the Tukey test (*P* ≤ 0.05). The columns described correspond to the means from six repetitions and the standard error (SE).

The data show that Si fertilization significantly increased the accumulation of SS concentration in plants exposed to NIP by approximately 120% in 2015 (**Figure 3A**) and by 180% in 2016 (**Figure 3B**), between the 0- and 10-mM SiK^®^ treatments, as previously observed by other authors in chestnut plants (Carneiro-Carvalho et al., 2020; Carneiro-Carvalho et al., 2021). When examining the IP, a similar tendency was observed. The Si-treated plants recorded a higher amount of SS than the control plants, 9.59 mg g^−1^ FM in 2015 (**Figure 3A**) and 7.80 mg g^−1^ FM in 2016 (**Figure 3B**) in the 10-mM SiK^®^ treatment, while the 0-mM SiK^®^ registered 4.10 mg g^−1^ FM in 2015 (**Figure 3A**) and 2.90 mg g^−1^ FM in 2016 (**Figure 3B**).

Comparing NIP with IP, the results show a decrease in the SS content under water deficit conditions. One explanation for these findings may be that drought induces the reduction of the catabolism of SS, as observed in previous studies in wheat plants (Zhang et al., 2013).

**Figure 4:**
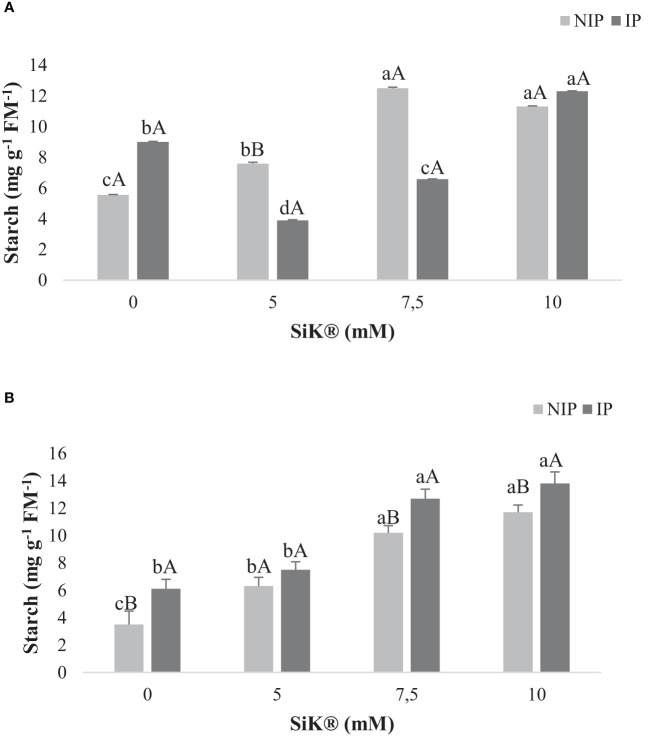
The effect of Si application (0, 5, 7.5, and 10 mM of SiK^®^) on the starch content (mg g^−1^ FM^−1^) of chestnut plants exposed to the non-irrigation phase (NIP) and the irrigation phase (IP) in 2015 **(A)** and 2016 **(B)**. Different uppercase letters indicate significant differences between treatments, and lowercase letters indicate significant differences between the same treatment at different water availability (NIP/IP), according to the Tukey test (*P* ≤ 0.05). The columns described correspond to the means from six repetitions and the standard error (SE).

Examination of the ST followed a similar trend as for the SS level. The untreated plants showed a lower ST content when compared with the Si-treated plants, which presented an increase in the amount of these metabolites when chestnuts were submitted to water stress (**Figure 4**).

### 3.3 Electrolyte leakage, lipid peroxidation, and hydrogen peroxide

ELP has been recommended as a valuable criterion for the identification of stress-resistant plants in several crop species (Pei et al., 2010).

**Figure 5:**
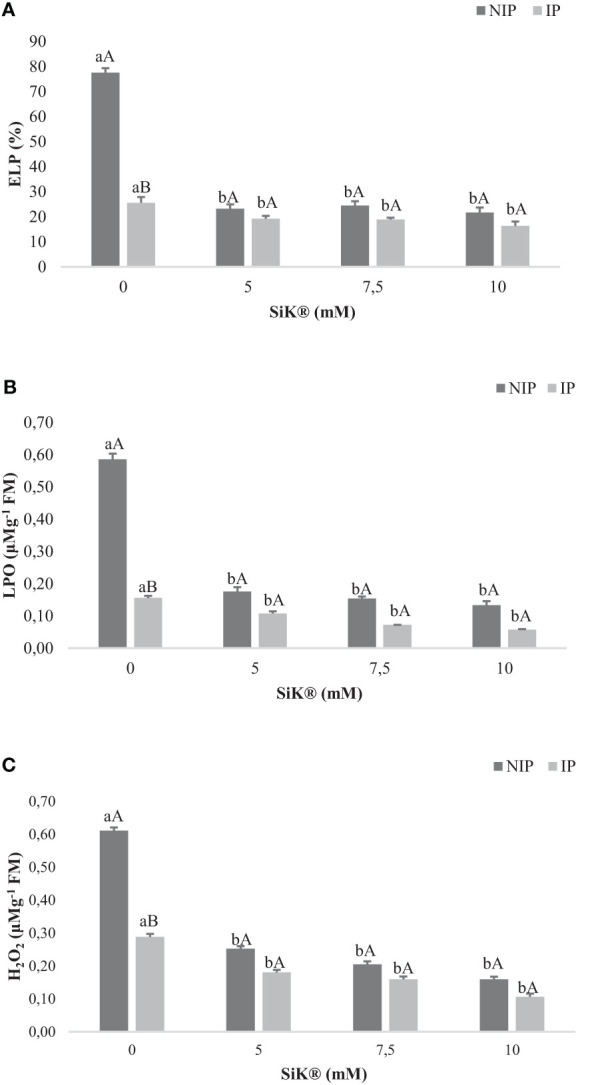
The effect of Si application (0, 5, 7.5, and 10 mM of SiK^®^) relative to **(A)** electrolyte leakage percentage (ELP), **(B)** lipid peroxidation (LPO), and **(C)** hydrogen peroxide (H_2_O_2_) sequentially exposed to a non-irrigation phase (NIP) and an irrigation phase (IP) in 2016 **(B)**. **(A)** ELP (%), **(B)** LPO (µM g^−1^ FM), and **(C)** H_2_O_2_ (µM g^−1^ FM). Different uppercase letters indicate significant differences between treatments, and lowercase letters indicate significant differences between the same treatment at different levels of water availability (NIP/IP), according to the Tukey test (*P* ≤ 0.05). The columns described correspond to the means from six repetitions and the standard error (SE).

Concerning the ELP measurements, it was observed that Si application in chestnut plants significantly reduced the ELP from 77.5% in 0 mM of SiK^®^ to 21.5% in 10 mM of SiK^®^, a decrease of approximately 260% (**Figure 5A**). The data show that Si-treated plants under IP present lower values of ELP (16.3%) compared with untreated plants (25.6%) (**Figure 5A**). When comparing NIP with IP (**Figure 5A**), the results suggest that Si application protected the lipid membranes from water stress; therefore, no significant differences were observed in Si-treated plants, consequently preventing oxidative damage.

The LPO in biological membranes is one of the main symptoms of oxidative stress in plants (Labbuda, 2013). The NIP induced a significant increase in LPO, which was higher in control plants, 0.585 µM g^−1^FM, than in Si-treated plants, 0.154 and 0.134 µM g^−1^ FM in the 7.5- and 10-mM SiK^®^ treatments, respectively (**Figure 5B**). When comparing NIP with IP, the results indicate that Si fertilization in chestnut plants promotes lower values of LPO under water stress since no significant differences were recorded between both phases.

The higher production of ROS by plants promotes oxidative stress, affecting the metabolism and resulting in extensive cellular damage and death (Ishibashi et al., 2011; Ediga et al., 2013). The study whoed that Si application reduced the H_2_O_2_ in NIP from 0.61 to 0.16 μM g^−1^ FM between 0 and 10 mM of SiK^®^, approximately 281%. In IP, once again, the Si application recorded lower values of H_2_O_2_, 0.16 and 0.10 µM g^−1^ FM (**Figure 5C**) in 7.5 and 10 mM of SiK^®^ than in control plants (0 mM of SiK^®^), 0.29 µM g^−1^ FM (**Figure 5C**). When comparing NIP with IP, the results indicate that Si application prevents the accumulation of ROS inside the tissues, protecting plants from oxidative damage. Results showed that the association of drought with the application of a higher concentration of Si (10 mM of SiK) led to a significant decrease in membrane damage (LPO, ELP and H_2_O_2_).

### 3.4 Proline content

**Figure 6:**
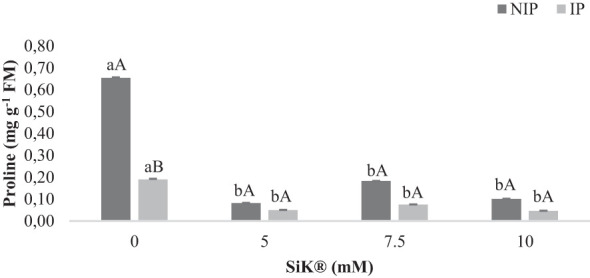
The effect of Si application (0, 5, 7.5, and 10 mM of SiK^®^) on the amount of proline (mg g^−1^FM) in chestnut plants exposed to the non-irrigation phase (NIP) and the irrigation phase (IP). Different uppercase letters indicate significant differences between treatments, and lowercase letters indicate significant differences between the same treatment at different levels of water availability (NIP/IP), according to the Tukey test (*P* ≤ 0.05). The columns described correspond to the means from six repetitions and the standard error (SE).

Proline (Pro) is an important osmoprotector and biomarker of abiotic stress (AlKahtani et al., 2021). Under the NIP, the synthesis of Pro increases significantly in control plants (0 mM of SiK^®^), 0.65 mg g^−1^ FM more than Si-treated plants, which present lower values, 0.18 and 0.10 mg g^−1^ FM (7.5 and 10 mM of SiK^®^, **Figure 6**). These findings reinforce the idea that the presence of Si inside chestnut plants promoted a better defense response against drought stress. This difference between Si-treated plants and control plants represents a reduction of 261% and 550%, respectively, in the 7.5- and 10-mM SiK^®^ treatments (**Figure 6**).

When comparing the results between NIP and IP, no significant differences were observed in Si-treated plants, while an increase of approximately 241% was observed in the Pro amount in the control plants (**Figure 6**). Moreover, the **Figure 6** suggest that Si fertilization in chestnut plants increases drought tolerance by limiting the accumulation of Pro in tissues under water deficit. There was a positive correlation between this parameter and the lower values of LPO and H_2_O_2_ contents (**Figures 5B, C**) in all Si-treated plants, demonstrating the higher tolerance of these plants to water deficit conditions.

### 3.5 Thiol content assay

The most prominent thiol in plants is the tripeptide glutathione compound (GSH), which has redox properties and is involved in the stress responses of plants (Ishibashi et al., 2011). **Table 1** shows a higher thiol (TL) synthesis in Si-treated plants 52% more (7.5 mM of SiK^®^) and 68% more (10 mM of SiK^®^) than in control plants (0 mM of SiK^®^) (**Table 1**). A similar tendency was observed in the IP, where the Si-treated plants exhibited a higher amount of TL, with this increase being proportional to the concentration of SiK^®^ applied, 0.125, 0.149, and 0.158 µmol g^−1^ DM on 5, 7.5, and 10 mM of SiK^®^ in plants, respectively, while the control plants presented only 0.103 µmol g^−1^ DM (**Table 1**).

**Table 1:** Effect of Si fertilization (0, 5, 7.5, and 10 mM of SiK^®^) on the total amount of thiol (mg g^−1^ DM) in the exposure of chestnut plants to the non-irrigation phase (NIP) and the irrigation phase (IP).

Si fertilization	NIP	IP
0 mM of SiK^®^	0.177 ± 0.005 bA	0.103 ± 0.09 bA
5 mM of SiK^®^	0.257 ± 0.007 aA	0.125 ± 0.007 bB
7.5 mM of SiK^®^	0.269 ± 0.004 aA	0.149 ± 0.008 aB
10 mM of SiK^®^	0.297 ± 0.002 aA	0.158 ± 0.008 aB

Data corresponded to the means from six repetitions and the standard error (SE). Different uppercase letters indicate significant differences between treatments, and lowercase letters indicate significant differences between the same treatment at different water available (NIP/IP) according to the Tukey test (P ≤ 0.05).

### 3.6 Quantification of soluble protein content

**Figure 7:**
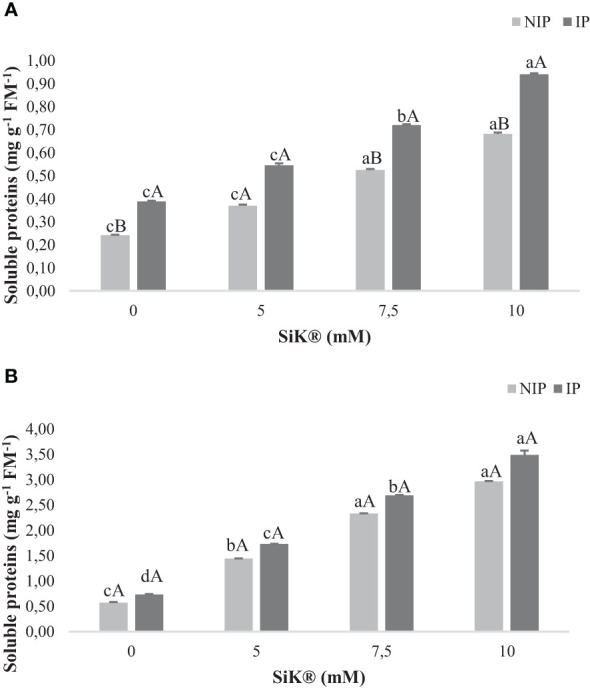
The effect of Si application (0, 5, 7.5, and 10 mM of SiK^®^) on the amount of soluble proteins (mg g^−1^ FM^−1^) in chestnut plants exposed to the non-irrigation phase (NIP) and the irrigation phase (IP) in **(A)** 2015 and **(B)** 2016. Different uppercase letters indicate significant differences between treatments, and lowercase letters indicate significant differences between the same treatment at different levels of water availability (NIP/IP), according to the Tukey test (*P* ≤ 0.05). The columns described correspond to the means from six repetitions and the standard error (SE).

Concerning the SP content, data indicate a substantial production of these metabolites in NIP by 180% (2015, **Figure 7A**) and 418% (2016, **Figure 7B**) when comparing the Si-treated plants (10 mM of SiK^®^) with the untreated plants (0 mM of SiK^®^). Consistently, in the IP, the Si-fertilized plants (10 mM of SiK^®^) show the highest amount of SP, approximately 143% (**Figure 7A**) in 2015 and 375% (**Figure 7B**) in 2016, in comparison with the control plants (0 mM of SiK^®^).

When comparing NIP with IP, **Figures 7A, B** show that the Si application promotes the increase in the amount of SP, from 0.681 to 0.940 mg g^−1^ FM^−1^ in 2015 and from 2.970 to 3.490 mg g^−1^ FM^−1^ in 2016, in the 10-mM SiK^®^ treatment.

### 3.7 Antioxidant activity

**Figure 8:**
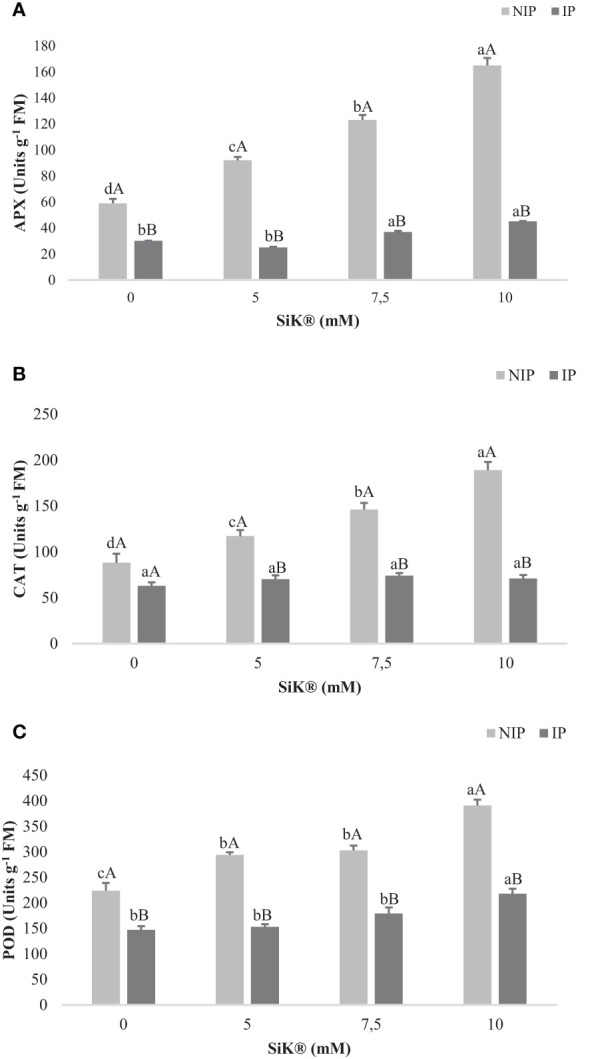
The effect of Si application (0, 5, 7.5, and 10 mM of SiK^®^) on **(A)** APX (units g^−1^ FM), **(B)** CAT (units g^−1^ FM), and POD **(C)** (units g^−1^ FM) in chestnut plants exposed to the non-irrigation phase (NIP) and the irrigation phase (IP). Different uppercase letters indicate significant differences between treatments, and lowercase letters indicate significant differences between the same treatment at different levels of water availability (NIP/IP), according to the Tukey test (*P* ≤ 0.05). The columns described correspond to the means from six repetitions and the standard error (SE).

The maximum increase in the APX activity was 165 nmol mg^−1^ protein min^−1^ achieved by the 10-mM SiK^®^ treatment, while the minimum value was 59 nmol mg^−1^ protein min^−1^ (**Figure 8A**), recorded by the control plants (0 mM of SiK^®^). The data suggest that Si fertilization promotes a significant increase in APX activity from 59 to 165 nmol mg^−1^ protein min^−1^, 180% more between the 0- and 10-mM SiK^®^ treatments (**Figure 8A**). During the IP, the values of APX registered were lower than for the NIP. However, the Si-treated plants presented 45 nmol mg^−1^ protein min^−1^ (10 mM of SiK^®^), while the control plants presented only 30 nmol mg^−1^ protein min^−1^ (0 mM of SiK^®^) (**Figure 8A**).

Consistently, CAT activity increased proportionally to the amount of SiK^®^ applied, 117, 146, and 189 nmol mg^−1^ protein min^−1^ (**Figure 8B**) in the 5-, 7.5-, and 10-mM treatments, while the untreated plants (0 mM of SiK^®^) presented only 88 nmol mg^−1^ protein min^−1^ (**Figure 8B**).

Regarding the POD activity, the Si-treated plants showed higher values of 303 nmol mg^−1^ protein min^−1^ (**Figure 8C**), approximately 200% and 279% more in 7.5 and 10 mM of SiK^®^, respectively, while the untreated plants (0 mM of SiK^®^) recorded only 104 nmol mg^−1^ protein min^−1^ (**Figure 8C**).

The present results show significant differences observed between the Si-treated plants and the control plants during the IP. Comparing the NIP with the IP, the results indicate that water stress significantly induces the antioxidant activity of the enzymes under study (APX, CAT, and POD) (**Figure 8**) in Si-treated plants under water stress, suggesting that Si application promotes the biochemical activity as a defense response to oxidative damage.

## 4 Discussion

Climate change is the major problem that threatens agricultural production in the 21st century, and increasing temperatures, decreasing rainfall, and drought stress are the major serious threats to the security of food worldwide. Abiotic stress negatively affects the development and yield of plants and produces unfavorable changes in the morphological, physiological, and biochemical systems. Si appears to be a solution for agriculture, being defined as an antistress element that ameliorates the impacts by regulating diverse functionalities on enhancing the growth and development of crops and modulating several metabolites during drought conditions (AlKahtani et al., 2021; Mir et al., 2022; Ahsan et al., 2023). The present study showed that silicon application can intensify the accumulation of secondary metabolites and the TP amount in chestnut leaves (**Figure 2**) as a biochemical defense mechanism against water stress, improving the antioxidant activity and consequently helping the plants to have more tolerance against water deficit than the untreated plants (0 mM of SiK^®^). The phenolic compounds are a vast class of secondary metabolites performing several roles for the defense and survival of plants. These compounds have a strong antioxidant capacity to protect cellular structures from oxidative damage and eliminate ROS from cells, forming an important first line of defense to deal with stress in different species (Zanetti et al., 2016; Kovács et al., 2022; Rocha et al., 2022). Similar results were found in soybean (Ediga et al., 2013), cacao (Rocha et al., 2022), and *Panicum maximum* plants (Quan et al., 2017). When comparing the NIP with the IP, the data indicate that Si-treated plants increased TP levels under stress conditions compared with non-stressed plants. TP has been described as a marker of abiotic stress tolerance in plants which present several phenolic compounds involved in oxidative stress caused by ROS, with the higher TP amount being a biochemical response to adapt to adverse environmental changes (Ashraf and Foolad, 2007). Several authors explained that the higher SS content (**Figure 2**) in Si-treated plants protects against ROS damage, conferring protection to the membrane integrity and promoting the stabilization of proteins/enzymes (Al-Mayahi, 2016; Verma et al., 2021). The data showed that Si application induced a higher accumulation of SS in the NIP (**Figure 3**) compared with control plants (0 mM of SiK^®^). The SS can act as osmotic agents and osmoprotectors to stabilize proteins and membranes in the osmotic adjustment production. This study suggests that Si fertilization promoted the increase in the ST amount in chestnuts under drought stress, corroborated by previous studies in cacao (Zanetti et al., 2016) and *Phoenix dactylifera* plants (Al-Mayahi, 2016). The high ST content (**Figure 4**) in Si-supplied plants under water deficit may be used as a reserve for the metabolic processes.

Relative to the membrane injury, the present study also showed a significant decrease in ROS in Si-treated chestnut plants under abiotic stress conditions similar to previous studies (Carneiro-Carvalho et al., 2020; Carneiro-Carvalho et al., 2021). In this study, oxidative damage was significantly reduced in *C. sativa* plants under water stress. When comparing the NIP with the IP, there was no significant difference between Si-treated plants (**Figures 4A, B**). One possible explanation can be that Si application helps limit the amount of ELP (**Figure 5A**), LPO (**Figure 5B**), and H_2_O_2_ (**Figure 5C**) under stress conditions in comparison with control plants. The data indicated that Si promotes the stabilization of the plasma membrane, allowing the osmotic adjustment and the decline of the oxidative damage of functional molecules, compared with control plants, and similar results were verified in Mandarin trees (Berríos et al., 2023) under water deficit. These findings are similar to previous studies, which referred that Si treatment can maintain the stability and functioning of plasma membranes, by the increase in the antioxidant defenses in maize (Afari et al., 2015) and cucumber plants (Zhu et al., 2004). In addition, the present study demonstrated that Si application reduces the accumulation of H_2_O_2_ in tissues and also influences the level of LPO (**Figure 5B**). Studies in fava bean also demonstrated that Si regulates the mechanisms for cellular redox homeostasis, promoting the transformation of superoxide to H_2_O and O_2_ by enhancing the activities of the H_2_O_2_-scavenging enzymes and conferring protection to the plants against oxidation damage (Abu-Muriefah, 2015). The data are coherent with the lower values of ELP (**Figure 5A**) and LPO (**Figure 5B**), demonstrating that Si fertilization acts as a defense mediator in NIP, protecting cells from oxidative stress by improving the efficiency of defense response systems, due to the promotion of the antioxidant activity. Similar results were reported in wheat (Verma et al., 2021), cotton (Farooq et al., 2013), and peach plants (Nascimento-Silva et al., 2023). This study further confirms that Si application in chestnut plants was positively correlated with the reduction in membrane damage (**Figure 5**) and the enhancement in enzyme defense activity (**Figure 8**).

Proline is considered a biochemical indicator of stress, and the results indicate that Si-treated plants significantly reduced the Pro content during the NIP (**Figure 6**). These results are corroborated by the lower values of ELP, LPO, and H_2_O_2_ (**Figure 5**) levels in the NIP, indicating that Si has a beneficial role in mitigating/reducing the negative effects of water stress. Similar results were found by other researchers, who reported the addition of Si in sunflower (Gunes et al., 2008) and olive plants (Radhi et al., 2021; Hassan et al., 2022). Therefore, the lower production of Pro content could be an advantage to saving more energy to increase the tolerance of plants against environmental stresses.

The application of Si is known to arbitrate biochemical processes to enhance biomass resilience under water deficit in plants (Mello et al., 2022). Similarly, in the current study, Si-augmented TL amount is higher in Si-treated plants, 7.5 and 10 mM of SiK^®^ (**Table 1**), than in control plants under water deficit conditions, suggesting that the presence of Si inside the plants can promote the synthesis of TP as a defense response. These results are corroborated by previous studies (Verma et al., 2021; Rocha et al., 2022), which reported that the Si application in plants under water stress stimulates the TL amount, e.g., glutathione (GSH) and cysteine (Cys), in grasses. The TL plays a central part in plant stress response by neutralizing the toxicity of overproduced ROS resulting from the water deficit (Silva, 2022). Concerning the content of SP, results show that Si fertilization promotes the protection of these biomolecules against NIP (**Figure 7**), reducing the ELP, LPO, and H_2_O_2_ amounts (**Figure 5**). Moreover, the lower values of SP content recorded by the control plants can be associated with the increase in Pro and the higher oxidative damage under water stress, resulting in destruction or a decrease in the biosynthesis of proteins, which can affect the membrane fluidity and structure in wheat, *Medicago sativa*, *Medicago arborea*, and *Alborea* plants (Kumar et al., 2017; Tani et al., 2019).

APX, CAT, and POD are antioxidant enzymes associated with the primary role in H_2_O_2_ detoxification, playing an essential protective role against water scarcity. The present study demonstrated that Si fertilization significantly increases APX, CAT, and POD activity during the NIP, with an increase of 179%, 189%, and 279% in the 10-mM SiK^®^ treatment (**Figure 8**) of these antioxidant enzymes involved in the resilience against drought. The present findings suggest that Si-treated plants were more resistant to water stress compared with untreated plants. This was attributed to the mitigation of oxidative damage and lower ELP, LPO, and H_2_O_2_ contents (**Figure 5**), promoted by Si fertilization, which can be associated with the increase in APX, CAT, and POD activity (**Figure 8**). These are considered important anti-drought mechanisms to control water deficit, corroborating the previous studies on oilseed rape plants (Abedi and Pakniyat, 2010). Early reports on apple (Abd El-Rhman et al., 2020), peach (Silva, 2022), and fig trees (Hussien and Kassem, 2021) proposed a rise in antioxidant activity in Si-treated plants during drought stress. The present findings are reinforced by previous studies that report a beneficial effect of Si on the increase of APX, CAT, and POD activity in response to drought stress in faba bean (Abu-Muriefah, 2015) and *P. dactylifera* (Al-Mayahi, 2016). This study suggests that Si fertilization in chestnut plants under drought stress could significantly enhance the defense capacity against oxidative damage, leading to a significant improvement in the biochemical defense system.

## 5 Conclusions

This research shows that Si can increase resilience against water stress and can improve the plant’s ability to recover from water stress after a period of drought. Effectively, fertilization with Si can be a promising and accessible strategy for non-irrigated groves to maintain their water status during warmer periods, which are increasingly frequent with the worsening of climate change. So, the results obtained bring many new data showing the beneficial effect of Si on the chestnut tree constituting the innovation of this study. Indeed, it was observed that Si promotes the resilience of chestnut trees to water stress, through the synthesis of biochemical compounds, reduction of oxidative stress in plants, increased activity of antioxidant enzymes, and the survival of plants. This is extremely important information for chestnut fruit producers. Having evaluated all the treatments under study, the recommended Si concentration is 10 mM, as it was observed that this concentration provides greater protection to chestnut trees during the water stress phase and faster recovery from this adverse situation physiology.

## Data availability statement

The raw data supporting the conclusions of this article will be made available by the authors, without undue reservation.

## Author contributions

AC-C- study concept, data collection, analysis and interpretation of results, write the manuscript and draft manuscript preparation. TP, JG-L, and RA study the concept and design and provided suggestions for the paper. RA and TP drafted the manuscript preparation and helped make the corrections the reviewers suggested. All authors contributed to the article and approved the submitted version.
